# Foil Strain Gauges Using Piezoresistive Carbon Nanotube Yarn: Fabrication and Calibration

**DOI:** 10.3390/s18020464

**Published:** 2018-02-05

**Authors:** Jandro L. Abot, Mário R. Góngora-Rubio, Jude C. Anike, César Y. Kiyono, Luis A. M. Mello, Valtemar F. Cardoso, Reinaldo L. S. Rosa, Derek A. Kuebler, Grace E. Brodeur, Amani H. Alotaibi, Marisa P. Coene, Lauren M. Coene, Elizabeth Jean, Rafael C. Santiago, Francisco H. A. Oliveira, Ricardo Rangel, Gilles P. Thomas, Kalayu Belay, Luciana W. da Silva, Rafael T. Moura, Antonio C. Seabra, Emílio C. N. Silva

**Affiliations:** 1 Department of Mechanical Engineering, The Catholic University of America, 620 Michigan Ave. NE, Washington, DC 20064, USA; 09anike@cua.edu (J.C.A.); 69kuebler@cua.edu (D.A.K.); 31brodeur@cua.edu (G.E.B.); amanialthqil@gmail.com (A.H.A.); 90coene@cua.edu (M.P.C.); coene@cua.edu (L.M.C.); jeanel@students.trinitydc.edu (E.J.); 2 Institute of Technological Research, Bionanomanufacturing Group, Av. Prof. Almeida Prado, 532, São Paulo SP-05508-901, Brazil; gongoram@ipt.br (M.R.G.-R.); guga.mello@gmail.com (L.A.M.M.); swluciana@ipt.br (L.W.d.S.); 3 Department of Mechatronics Engineering and Mechanical Systems, School of Engineering, University of São Paulo, Av. Prof. Mello Moraes, 2231, São Paulo SP-05508-900, Brazil; ckiyono@gmail.com (C.Y.K.); rafael.santiago@usp.br (R.C.S.); francisco.fo78@gmail.com (F.H.A.O.); gillespierre.thomas@gmail.com (G.P.T.); moura.gmsie@usp.br (R.T.M.); ecnsilva@usp.br (E.C.N.S.); 4 Department of Electrical Engineering, School of Engineering, University of São Paulo, Av. Prof. Luciano Gualberto, 158, São Paulo SP-05508-010, Brazil; valtema@gmail.com (V.F.C.); reilucasrosa@gmail.com (R.L.S.R.); rrangel@lsi.usp.br (R.R.); antonio.seabra@usp.br (A.C.S.); 5 Department of Physics, Florida Agricultural and Mechanical University, 2077 East Paul Dirac Dr., Tallahassee, FL 32310, USA; kalayu.belay@famu.edu

**Keywords:** carbon nanotube yarn, strain gauge, piezoresistive sensor, micro-fabrication, experimental characterization

## Abstract

Carbon nanotube yarns are micron-scale fibers comprised by tens of thousands of carbon nanotubes in their cross section and exhibiting piezoresistive characteristics that can be tapped to sense strain. This paper presents the details of novel foil strain gauge sensor configurations comprising carbon nanotube yarn as the piezoresistive sensing element. The foil strain gauge sensors are designed using the results of parametric studies that maximize the sensitivity of the sensors to mechanical loading. The fabrication details of the strain gauge sensors that exhibit the highest sensitivity, based on the modeling results, are described including the materials and procedures used in the first prototypes. Details of the calibration of the foil strain gauge sensors are also provided and discussed in the context of their electromechanical characterization when bonded to metallic specimens. This characterization included studying their response under monotonic and cyclic mechanical loading. It was shown that these foil strain gauge sensors comprising carbon nanotube yarn are sensitive enough to capture strain and can replicate the loading and unloading cycles. It was also observed that the loading rate affects their piezoresistive response and that the gauge factors were all above one order of magnitude higher than those of typical metallic foil strain gauges. Based on these calibration results on the initial sensor configurations, new foil strain gauge configurations will be designed and fabricated, to increase the strain gauge factors even more.

## 1. Introduction

Strains can be measured by sensors that rely on the piezoresistive effect [1,2,3,4,5,6,7,8,9,10,11,12,13,14,15,16,17,18,19,20,21,22,23,24,25,26,27,28,29,30,31,32,33], the frequency shift of a resonator’s fundamental mode [34,35,36], the piezoelectric effect [37,38], the capacitance change [39,40,41,42,43], the optical properties changes [44,45,46,47,48,49], and other effects [50,51,52]. Piezoresistive effects consist of changes in the electrical resistance of a material when subjected to a mechanical strain. By applying an electrical signal, these changes can be measured, and the strain can be determined through a calibration procedure. In piezoresistive strain gauges, the strain is related to the change in resistance. Metallic foil strain gauge sensors are used to measure strain in the surface of components or structures and typically capture strains of up to 5% with gauge factors that mainly depend on the reduction of the cross-sectional area and the elongation of the resistor and hover around 2 [1,2,3,4]. The most typical alloy used in these sensors is Constantan (55% copper and 45% nickel), which exhibits relatively high strain sensitivity, relative insensitivity to temperature, high enough resistivity to achieve measurable resistance values, good fatigue capabilities, and relatively high elongations. However, Constantan exhibits a permanent drift at temperatures above 65 °C, which could be a problem to achieve stable strain measurements over a period of hours or days.

Semiconductor strain gauges rely on the piezoresistive effect of silicon or germanium and measure changes in resistance with respect to applied stresses [5,6,7]. They are accurate, repeatable, and have a gauge factor that depends mostly on the effect of the piezoresistive part ranging between 200 and 500 according to the doping concentration and lattice orientation [7]. They may exhibit significant nonlinearity and higher temperature sensitivity [5] but hysteresis and creep could be reduced through material treatment techniques [7]. Other sensors rely on the piezoresistive feature of carbon fibers that constitute the reinforcement in composite materials although their piezoresistance is not tailorable and may be reduced by the significant number of fibers in typical composite structures [9,10]. Other piezoresistive strain sensors include carbon fibers in polymer matrices that can capture large strains when monitored by electrochemical impedance spectroscopy (EIS) at room temperatures [11], and conductive fabrics [12]. Silicon nanowires also exhibit high piezoresistivity and are also promising for strain sensing at the nanoscale level [13]. Other strain sensors rely on the piezoresistive nature of carbon nanotubes that are dispersed in polymeric matrices, forming nanocomposites, and exhibiting a quasi-linear resistance change-strain response with gauge factors varying between −200 and 500 [14,15,16,17,18,19,20,21,22,23]. More recently, strain gauges using carbon nanotubes, graphene and other carbon nanomaterials are being developed and offer the promise of high gauge factors [24,25,26,27,28,29,30,31,32,33].

Many of the commonly used strain sensors, especially fiber optic sensors, may alter the material’s microstructure and some may compromise its integrity by requiring many sensors and complex equipment to acquire the strain gauge data. The ideal strain gauge sensors should be smaller than the microstructure of the host material, low in cost, easy to integrate, highly sensitive to strain, insensitive to temperature variations, and not require complex or expensive measuring equipment. Consequently, nanoscale materials like nanowires and nanotubes, and new concepts for sensing at the nanoscale are very promising. However, integrating them throughout an entire structure is not easy to implement. Carbon nanotube (CNT) yarns are micro-scale fibers that contain thousands of intertwined carbon nanotubes in their cross sections and exhibit piezoresistance characteristics that can be tapped for sensing strain [53,54,55,56,57,58,59,60]. The use of strain gauge sensors comprising CNT yarn may offer a feasible and practical way to measure strain inside polymeric and composite materials [61,62,63,64], and on the surface of all materials [65]. The work presented in this paper focuses on foil strain gauge sensors that could be adhered to external surfaces.

Piezoresistivity-based foil strain gauge sensors are usually made of a piezoresistive membrane layer attached to a flexible substrate. This flexible structure acts as a compliant mechanism that translates an input force into local strain and stress in the piezoresistive layer so that changes in electrical resistivity can be monitored and correlated to strain using the piezoresistivity effect. The piezoresistive layer can be electrically connected to a Wheatstone bridge to improve the sensor’ sensitivity and compensate undesirable temperature effects [1]. Size, geometry and the relative arrangement of the piezoresistive membrane within the sensor significantly affect the performance of strain gauge sensors [66]. Typically, the strain gauge design objective is to obtain the location and configuration of the material that maximizes its sensitivity to external loading.

This paper summarizes the fabrication and calibration results on foil strain gauges comprising piezoresistive CNT yarn. Section 2 presents the foil strain gauge sensor concept and the piezoresistive response of the sensing element, i.e., the CNT yarn. Section 3 briefly describes the modeling results of the piezoresistive response of these foil strain gauge sensors that lead to the prototype selection. Section 4 describes the fabrication details of the foil strain gauge sensors. Section 5 describes the calibration of the foil strain gauge sensors and their gauge factors. Section 6 presents the conclusions of the study.

## 2. Sensor Concept and Its Sensing Element

**Figure 1:**
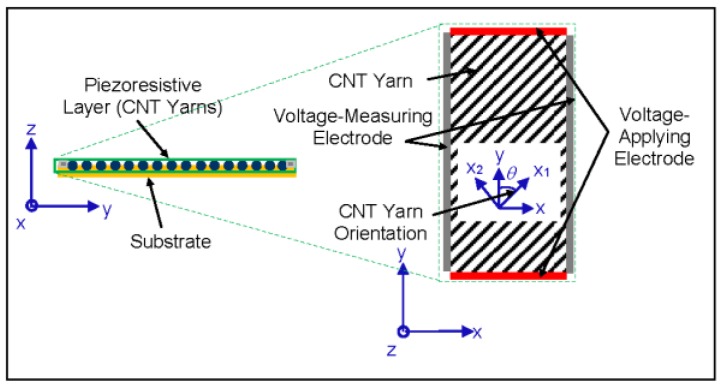
Schematic of cross-section of the foil strain gauge sensor comprising CNT yarns. Inset: top schematic view of the arrangement of the CNT yarns in a unidirectional configuration [65].

**Figure 2:**
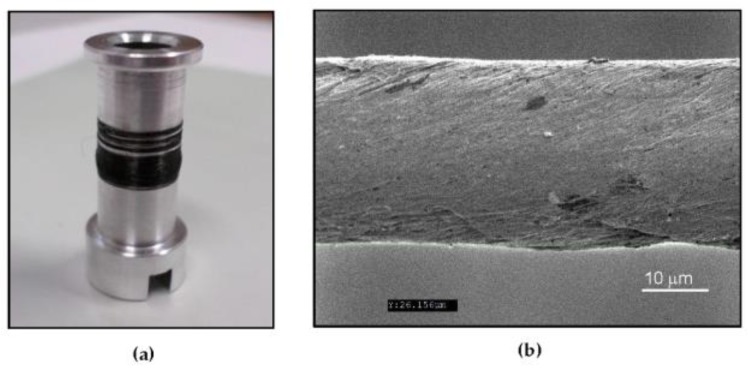
(**a**) Optical image of a spool with the CNT yarn; (**b**) Scanning Electron Microscope (SEM) image of a one-thread CNT yarn.

A schematic of the foil strain gauge sensor configuration including the piezoresistive layer containing the CNT yarns, the substrate composed of a polymeric material, and the electrodes is shown in Figure 1 [65]. The “building block” or basic initial configuration of the piezoresistive layer is an arrangement of parallel CNT yarns as shown in the inset of Figure 1. Other arrangements of the CNT yarns are also possible including bidirectional configurations [65]. The CNT yarns in this study were dry-spun from the sides of 400 μm-high vertically aligned arrays composed of carbon nanotubes consisting of 2 to 3 walls grown through water-assisted Chemical Vapor Deposition (CVD) [56,57,58]. The CNT yarns consist of a single thread with an approximate angle of twist of 30°. An optical image of the spool containing the CNT yarn is shown in Figure 2a and a Scanning Electron Microscopy (SEM) image of the one-thread CNT yarn is presented in Figure 2b. This CNT yarn has a diameter of about 30 microns with mechanical and electrical properties that are similar to those provided elsewhere [58]. Approximately, two thirds of the carbon nanotubes are semiconducting, and the other third are metallic. The CNT yarns were densified with acetone without altering the chemistry of the nanotubes. These one-thread yarns have a density of about 0.9 g cm^−3^ placing them in the very upper range of yarns’ densities with the corresponding implications in terms of higher uniaxial tensile elastic modulus and strength, and lower electrical resistivity [56]. These CNT yarns have a piezoresistive response that depends on the nanotubes’ geometry and chiralities, the twist angle of the yarn, and on several other yarn’s structure and loading parameters [62,67,68,69,70,71].

The electromechanical response of the unconstrained (free) CNT yarn used in this study had been previously determined using four probe measurements [67,68,69,70,71]. Based on previous experimental results, it is hypothesized that two underlying physical phenomena govern the electromechanical response of CNT yarns: (1) decrease in contact length of the carbon nanotube bundles as the CNT yarn is stretched during loading leading to a resistance increase and a converse increase in the contact length of the carbon nanotube bundles during unloading leading to a resistance decrease; and (2) a decrease in resistance due to inter-tube/inter-bundle slippage (inelastic shear motion) caused by yarn’s relaxation and structural reformation during the loading segments, and a continuous decrease in resistance during unloading as the yarn recovers its (conductive) structure. At high strain rates, the first phenomenon dominates during both loading and unloading [69,71]. In the case of lower strain rates, the second phenomenon dominates during loading and the first phenomenon dominates during unloading [69,71]. These hypotheses need confirmation through computational modeling.

## 3. Modeling of Piezoresistive Response of Foil Strain Gauges

A parametric model had been implemented in Matlab^TM^ to design four-terminal foil strain gauge sensors comprising piezoresistive CNT yarns (Figure 1) [65]. The sensitivity of the foil strain gauge sensors was calculated by changing several geometrical and material parameters including the shape and dimensions of the foil strain gauge sensors and the exerted load [65]. It was concluded that the highest sensitivity could be achieved in the case of a square sensor with CNT yarns oriented at 70° and spaced as close to each other as possible. The foil strain gauge sensor is sensitive to all tractions although the highest sensitivity is achieved when a normal traction is relatively aligned with the CNT yarn direction [65]. The dimensions of the sensor play an important role in the sensitivity although the actual configuration of the CNT yarns within the sensor and their relative location of the voltage-measuring electrodes are critical. The spacing factor, which is the normalized distance (ratio of distance between CNT yarns and the diameter of the CNT yarn) and the Poisson’s ratio of the CNT yarn play also an important role in the sensitivity of the foil strain gauge [65]. The lower the spacing factor and the higher the Poisson’s ratio, the higher the sensitivity. The modeling results indicate that the sensitivity of these foil strain gauge sensors is sufficient to measure strain and that their gauge factors could be one order of magnitude higher than those of metallic foil strain gauges [65].

## 4. Fabrication of Foil Strain Gauges

**Figure 3:**
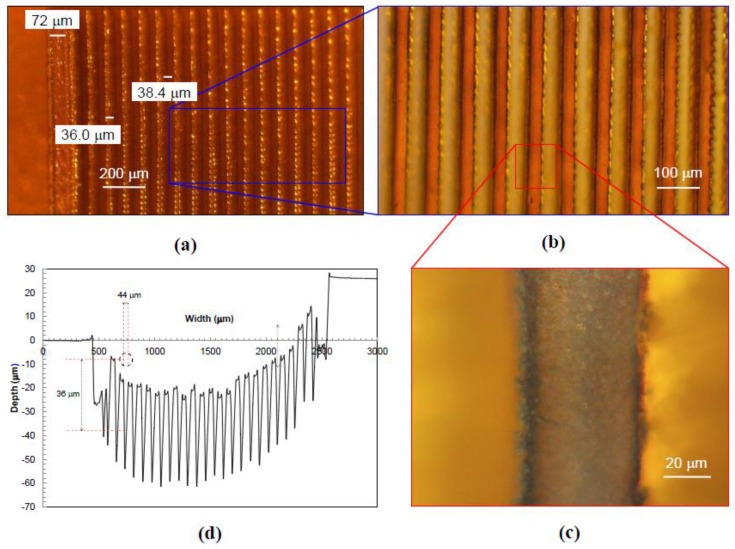
Foil strain gauge substrate with grooves oriented at 0°. (**a**) Optical image of substrate including one border for the electrode; (**b**) Close-up optical image of substrate; (**c**) Close-up optical image of a single groove; (**d**) Profilometer data indicating the width and depth of each groove.

**Figure 4:**
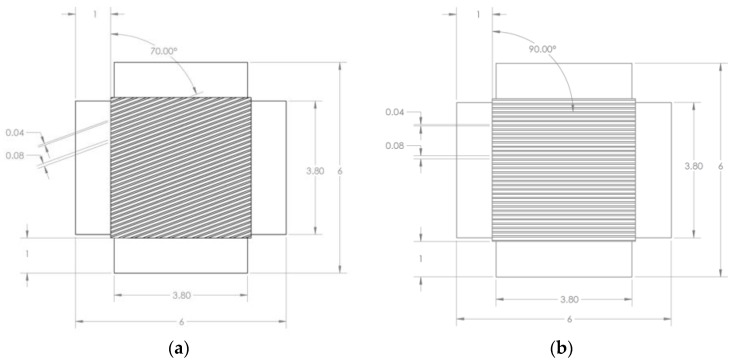
Schematics of the foil strain gauge substrates showing the grooves. (**a**) Grooves oriented at 70°; (**b**) Grooves oriented at 0° or 90°. Dimensions are in mm.

A set of square sensors, 2 mm by 2 mm, with CNT yarns spaced one diameter apart and arranged at 0° and 70° with respect to the loading direction was fabricated and used for calibration purposes. There were other sensor concepts that would yield higher sensitivity including rectangular shapes and bidirectional configurations but due to their complexity, only a set of simpler designs were fabricated initially. Kapton HN^TM^ polyimide films with a 125 μm-thickness were used as the substrate material and micro-channels (grooves) were created for the CNT yarns using a laser drilling technique to accommodate the CNT yarns. Images and drawings of the fabricated samples can be observed in Figure 3 and Figure 4, respectively. Several configurations for these micro-channels were considered including varying their width and spacing between them. The machining of the Kapton film to create the micro-channels was done using a rapid prototyping device, a laser circuit structuring machine (LPKF ProtoLaser U3). This equipment had its laser diode tuned to a wavelength of 355 nm, with a lower adjustable beam diameter of 10 µm, and a maximum power of about 6 W (typically at a laser pulse frequency of 40 kHz) reaching 30 mW µm^−2^. The machining is very rapid although the power of the laser tool needs to be tailored to the specific application (0.2 W in this case). Figure 3a–c show optical images of an initial configuration of the substrate with grooves oriented at 0°. Figure 3d includes surface profilometry results, which indicate the width and depth of the actual grooves (surface roughness was not investigated in this work). Figure 4a,b show the schematics of the substrate film with grooves oriented at 70° and 0°, which were the orientations ultimately chosen to fabricate the first foil strain gauge prototypes.

**Figure 5:**
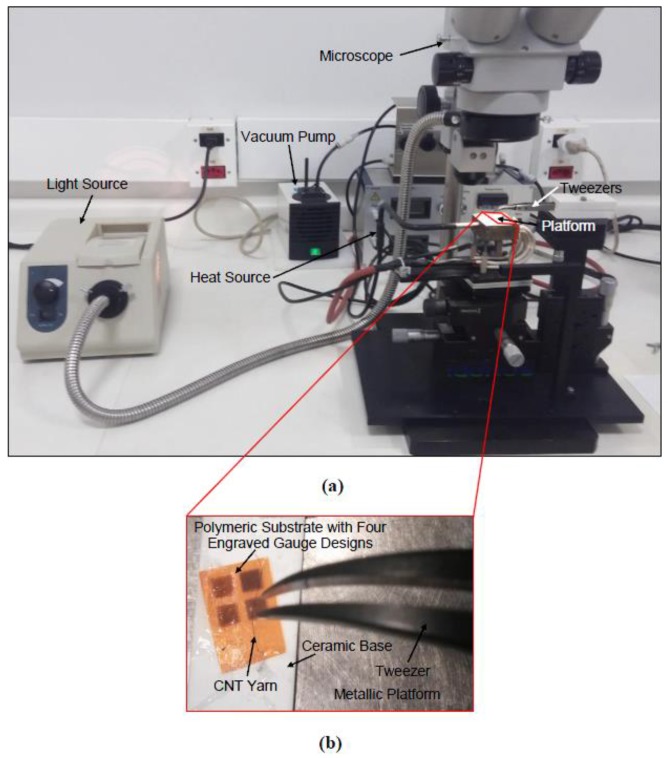
(**a**) Optical image of the experimental setup used initially to place the CNT yarns in the substrate consisting of a microscope with an adjustable platform and a vacuum connection; (**b**) Close-up optical image of the adjustable platform.

**Figure 6:**
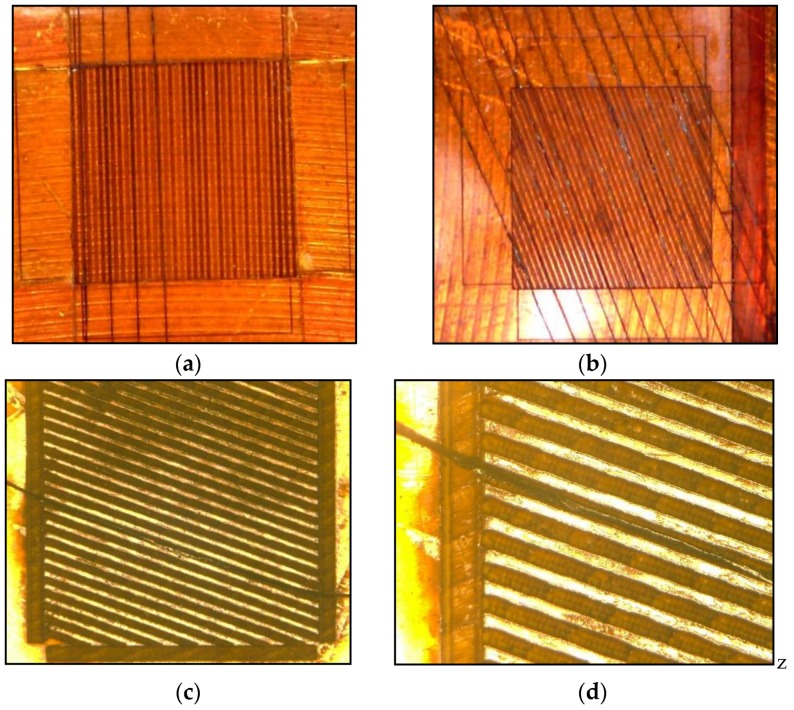

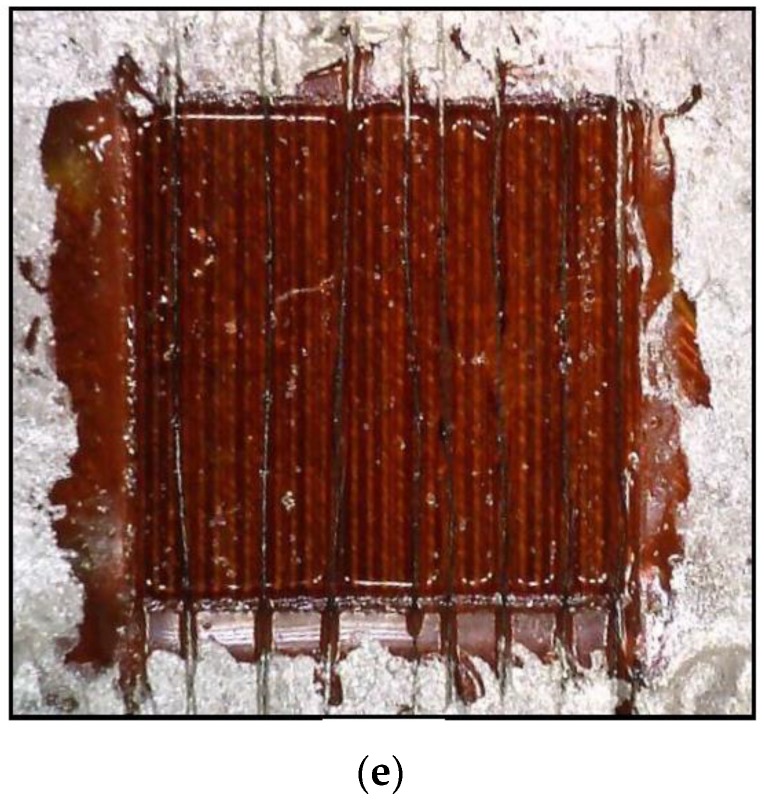
Optical images of the foil strain gauge prototypes: (**a**) Substrate with several CNT yarns placed in the grooves at 0°-inclination; (**b**) Substrate with several CNT yarns placed in the grooves at 70°-inclination. (**c**) Substrate with a CNT yarn placed in the groove; (**d**) Close-up of the substrate with a single CNT yarn placed in the groove; (**e**) Complete gauge showing CNT yarns in the grooves, adhesive layer on the gauge area, and conductive silver on the electrodes.

The placement of the CNT yarns in the substrate requires precision to ensure that the piezoresistive layer of the foilstrain gauge undergoes the same strain as the substrate layer that is bonded to the host material. Initially, a vacuum plate holding the machined substrate was used and the CNT yarns were then placed on each micro-channel using precision tweezers. The excess of the CNT yarns was cut using precision scissors. In the first foil strain gauge prototypes, one of every other groove were completed with CNT yarns. The experimental setup used to place the CNT yarns in the substrate initially is shown in Figure 5. To bond the CNT yarns to the substrate, an adhesive is applied to the substrate. Initially, a clear Araldite epoxy adhesive was used for an easier visualization of the CNT yarns within the substrate. Figure 6a,d show optical images of the CNT yarns in the substrate grooves made on the 125-μm-thick Kapton HN film. The final steps include filling of the border micro-channels with a conductive epoxy compound (Pelco^TM^, Clovis, CA, USA, high performance silver paste) to create the terminal electrodes.

The relative depth of the CNT yarns with respect to the substrate plays an important role in the piezoresistive response of the foil strain sensor. It is estimated that the CNT yarns were embedded about 80% deep into the substrate’s grooves. This configuration would allow the measurement of strain mostly in the longitudinal direction.

## 5. Calibration of Foil Strain Gauges

**Figure 7:**
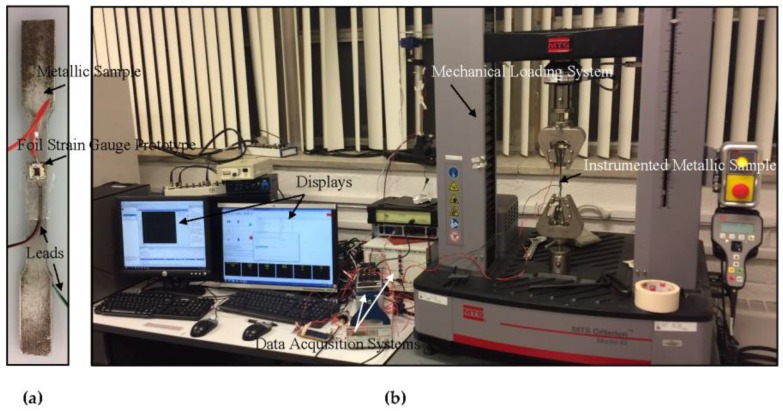
(**a**) Optical image of the dog-bone steel sample instrumented with a foil strain gauge prototype; (**b**) Experimental setup used to calibrate foil strain gauge prototypes.

An experimental program was designed to calibrate the foil strain gauge prototypes and to determine any potential piezoresistive hysteresis, relaxations and other material nonlinearities. The experimental setups to determine the electromechanical response of the fabricated foil strain gauge sensors and the corresponding results are presented and discussed next. The foil strain gauge prototype was bonded to a steel dog-bone sample using a cyanoacrylate-based adhesive (MBond 200 from Micro-Measurements, Raleigh, NC, USA). The dog-bone steel sample was 82 mm-long with transverse cross-section dimensions of 12.5 mm by 3.0 mm, respectively (Figure 7a). The sample was initially subjected to quasi-static tensile loading in a servo-hydraulic mechanical testing system (MTS Criterion 43 loading machine with a 30-kN load cell). The experiments were run under load control and the load was first increased monotonically and then cycled to achieve a maximum strain of approximately 0.4% at a displacement rate of 300 μm/min and an approximate strain rate of 5.5 × 10^−5^ s^−1^. This loading pattern was selected so that the steel sample stayed within their linear elastic regime. One to five cycles were used as the loading pattern and this number was deemed sufficient initially to carry out the strain gauge factor calculations and piezoresistive analysis. A metallic foil strain gauge (EA-06-125–120 from Micro-Measurements) was bonded on the exact same lengthwise location but the other side of the metallic sample to determine the strain directly on the sample and correlate it with that of the foil strain gauge prototype of this study. A National Instruments (NI, Austin, TX, USA) 4072 LCR (inductance-capacitance-resistance) card mounted on a NI-PXI 1033 chassis and an NI 9219 card mounted on a NI 9178 chassis were used to acquire the electrical data from the foil strain gauge prototypes and the metallic foil strain gauges. Finally, the data acquisition rates of the control software of both devices were set to approximately 10 Hz, and mechanical and electrical data acquisitions were triggered for immediate and simple correlation. An optical image of this experimental setup is presented in Figure 7b.

The electrical resistance is measured from the lateral electrodes (grey electrodes in Figure 1). Since the foil strain gauge in these calibration results consisted of the 0–90° configuration, only the two opposite electrodes in contact with the CNT yarns are used but the four electrodes could be used for all other configurations. The initial resistance in the first foil strain gauge prototypes was about 1.2 Ω, which corresponded to their initial resistance before the loading was applied. The resistance of the foil strain gauge prototype could also be estimated based on the resistance of each CNT yarn embedded in the grooves and their lengths using a model of resistors in parallel.

**Figure 8:**
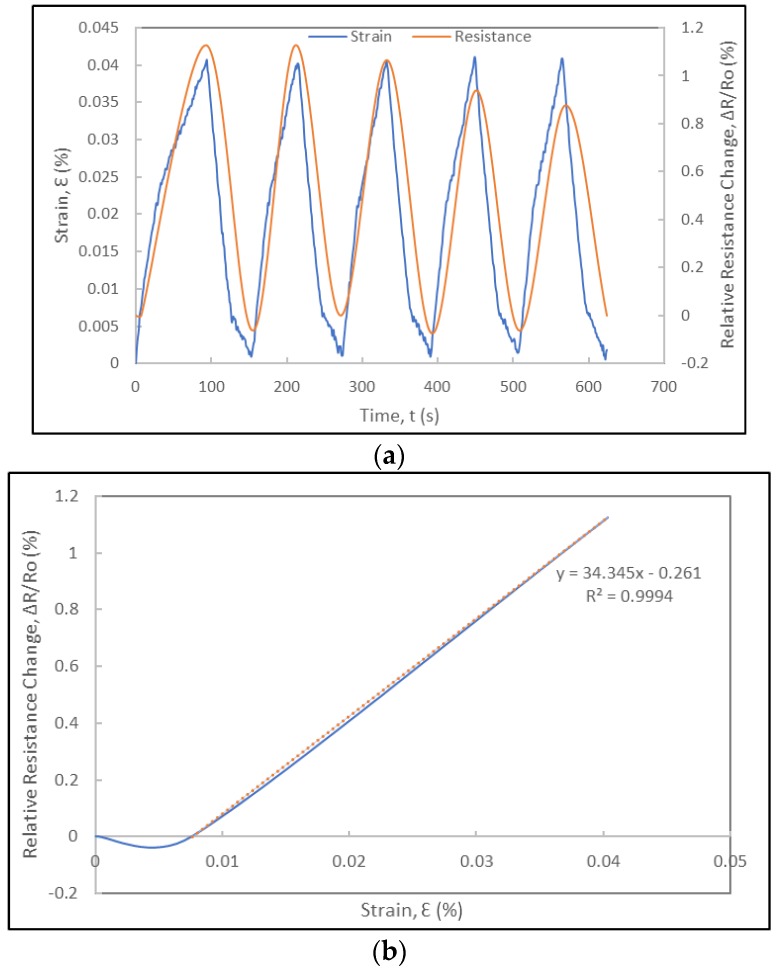
Electromechanical response of a foil strain gauge prototype under cyclic loading at a displacement rate of 300 µm/min: (**a**) Strain and relative resistance change histories during five loading-unloading cycles; (**b**) Relative resistance change versus strain curve of first loading cycle and corresponding gauge factor.

**Figure 9:**
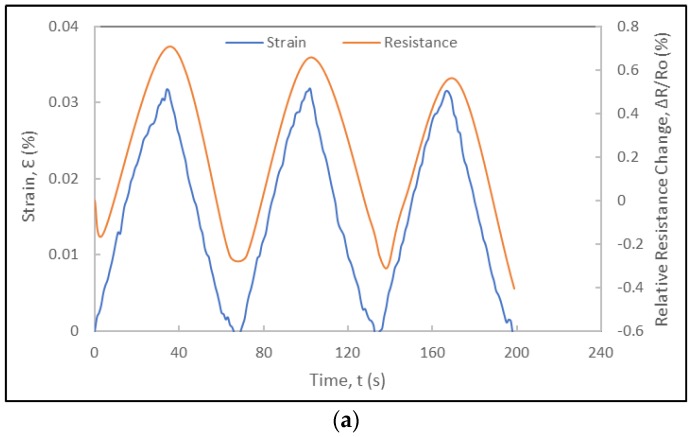

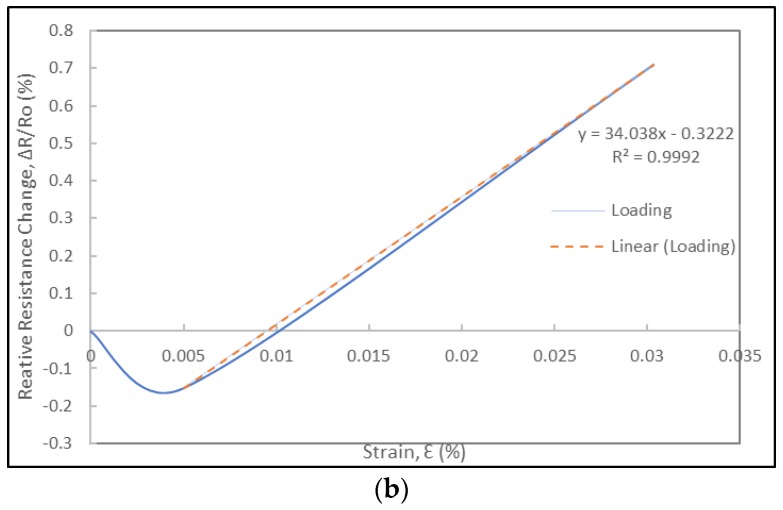
Electromechanical response of the same foil strain gauge prototype under subsequent cyclic loading at a displacement rate of 300 µm/min: (**a**) Strain and relative resistance change histories during three loading-unloading cycles. (**b**) Relative resistance change versus strain curve of first loading cycle and corresponding gauge factor.

**Figure 10:**
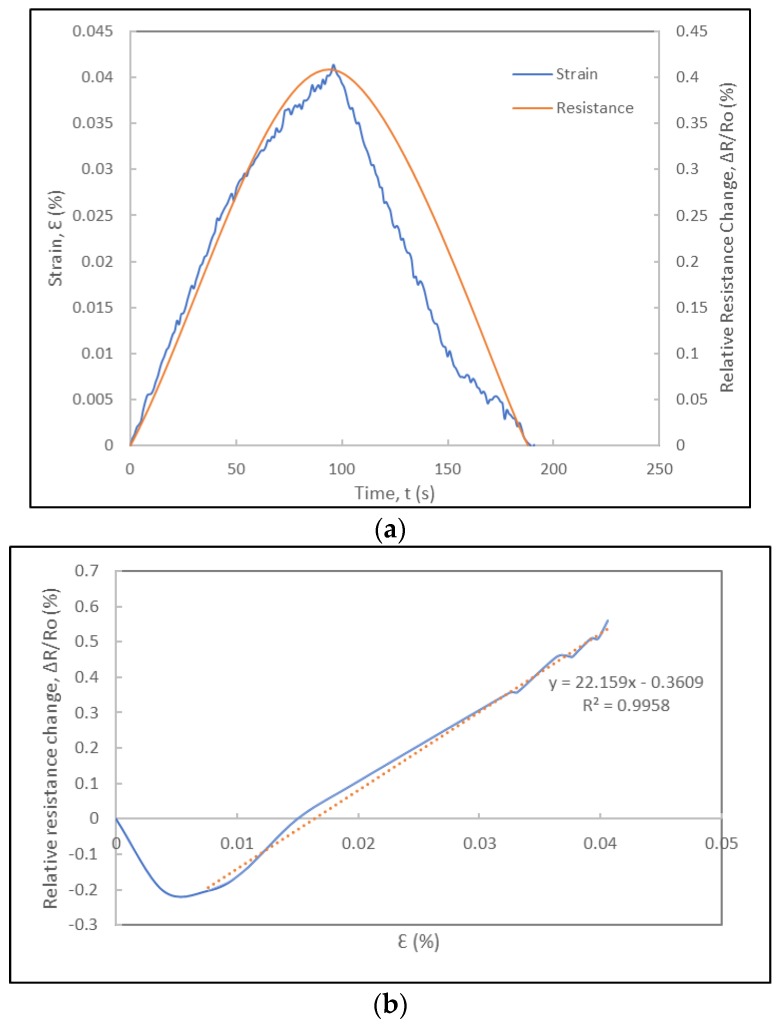
Electromechanical response of foil strain gauge prototype under cyclic loading at displacement rate of 150 µm/min: (**a**) Strain and relative resistance change histories during one cycle; (**b**) Relative resistance change versus strain curve of loading cycle and corresponding gauge factor.

The experimental results include the correlation of the strain data from the metallic foil strain gauge and the resistance data of the foil strain gauge prototype (Figure 8). Figure 8a shows the applied strain and relative resistance change histories. The relative resistance change data was noise-reduced and smoothened using digital non-linear filters and curve fitting models on Excel^TM^ and Matlab^TM^. The relative resistance change history curves indicate that the foil strain gauge sensor prototype is responsive to the loading and exhibits a cyclic response that mimics perfectly that of the applied strain history. The duration of each cycle is identical to that of the applied strain cycle (obtained from the strain history of the metallic foil strain gauge) and no lag in the response of the foil strain gauge is consistently observed in each cycle, as shown in Figure 8, Figure 9 and Figure 10, respectively. In the case of the experiments at 300 μm/min (5.5 × 10^−5^ s^−1^), the relative resistance change reaches about 1% for about 0.04%-strain levels. Towards their fourth cycles, a slight decrease in the piezoresistive response of the strain gauge prototype was observed in Figure 8. As the same foil strain gauge prototype was immediately subjected to additional cyclic loading (Figure 9), it was observed that the gauge would not recover completely exhibiting a non-zero resistance. Figure 10 shows the piezoresistive response of the foil strain gauge prototype subjected to a slower loading rate (150 μm/min or 2.8 × 10^−5^ s^−1^). It is observed one more time that the duration and peaks of the relative resistance change history match exactly that of the applied strain history.

Based on these preliminary results, design modifications to the foil strain gauges will be implemented and a new detailed calibration will be conducted including several higher strain levels and strain rates. Specifically, foil strain gauge configurations that include CNT yarns aligned with the loading direction, smaller separations between the CNT yarns and larger electrodes are expected to yield more robust sensors and lead to a better understanding of phenomena such as CNT yarn-adhesive interplay and other phenomena that may control their deformation.

The gauge factors of the strain gauge prototypes can be determined assuming uniaxial stress conditions and using the elastic properties of the sensing material [1]:

(1) $$GF=\frac{1}{\varepsilon }\frac{\Delta R}{R}=1+2\nu +\frac{1}{\varepsilon }\frac{\Delta \rho }{\rho }$$

where *R* is the resistance of the foil strain gauge, *ν* is the Poisson’s ratio of the foil strain gauge, *ε* is the longitudinal strain, and *ρ* is the electrical resistivity of the foil strain gauge. From Equation (1), it can be observed that the gauge factor includes contributions from three terms. In metallic foil strain gauges, the fractional change in electrical resistivity is almost insignificant compared to the dimensional effects given by the first two terms. In the case of CNT yarns, the gauge factors can be determined from the relative resistance change versus the strain curves under tension. These values vary depending on the strain rate, but they were determined to vary between 0.1 and 0.5 for the unconstrained CNT yarns [68,69,71] and between 1 and 30 for the CNT yarns integrated in polymeric materials [70,71]. For both unconstrained and constrained CNT yarns, the strain rate plays a very important role in their sensitivity with higher strain rates favoring higher gauge factors [70,71]. It is worth mentioning that in the case of the unconstrained CNT yarns and when the strain rates are very low (0.001 min^−1^ and below), there is a negative piezoresistance that leads to negative gauge factors [65,71]. These gauge factors are also influenced by the dimensional change when the Poisson’s ratio of the CNT yarn is high (6–8), which occurs at higher strain levels [72], and by the intertube resistance of carbon nanotubes either physically in contact (contact resistance) or with a gap between the nanotubes (tunneling resistance) [62,70]. The gauge factor of the free CNT yarn is a bit lower than that of metallic foil strain gauges and significantly lower than that of semiconductor silicon strain gauges [6,71]. However, the gauge factor of the constrained CNT yarns integrated in a polymer medium is significantly higher and one order of magnitude higher than that of the metallic foil strain gauges [70]. The foil strain gauges include CNT yarns constrained by grooves and a polymer, and thus they will more closely resemble the piezoresistive response of the constrained CNT yarns and their sensitivity. In this study, the slope of the relative resistance change versus strain curves in each cycle (either a loading or unloading segment) is used to determine the gauge factors. Figure 8b shows the curve for the loading segment of cycle 1. Using a linear regression, the gauge factor is determined to be approximately 34. Another experiment conducted on the same foil strain gauge prototype led to the same gauge factor as seen in Figure 9b. The loading rate and strain rate play a role in the sensitivity of the strain gauges and the gauge factors for a lower displacement rate (150 μm/min) was lower reaching a maximum value of 22 (Figure 10b).

The electromechanical characterization of the next generation of strain gauge prototypes will include obtaining more information about relaxation times especially as the number of cycles increases, and their dynamic strain bandwidth to determine if high enough to use them to measure most structural modes of vibration and impact events. The stability of strain gauges against temperature is critical to ensure acquiring the proper strain level. The temperature effects including the temperature coefficient resistance of these strain gauge prototypes will also be determined when calibrating future foil strain gauge prototypes.

## 6. Conclusions

Foil strain gauge sensor concepts comprising unidirectional and parallel carbon nanotube yarns are being developed and the first experimental results including their fabrication and calibration details are described in this paper. The modeling of the piezoresistive response of the strain gauges had indicated that their sensitivity would be enough to measure strain and that their gauge factors could potentially exceed those of metallic foil strain gauges. The first foil strain gauge prototypes comprising CNT yarns were fabricated considering the configurations that would provide the highest sensitivity according to the modeling effort. The calibration results indicate that these foil strain gauges were sensitive to the loading and that their resistance history correlated perfectly with the applied cyclic strain history, both in the peak and duration of each cycle. The gauge factors were determined to be consistently one order of magnitude higher than those of metallic foil strain gauges. This paper contents represent the initial experimental results on the development of foil strain gauges comprising piezoresistive CNT yarn, which may provide a more sensitive means to measure strains than existing technologies. Based on these results, new foil strain gauge prototype concepts will be fabricated and developed towards increasing robustness and sensitivities.
